# Supplementary material for: Content-rich biological network constructed by mining PubMed abstracts
Source: BMC Bioinformatics. 2004 Oct 8;5:147. doi: 10.1186/1471-2105-5-147 (PMC528731; doi:10.1186/1471-2105-5-147)
Supplement: Additional File 2 — The original results of the above study (non-essential files are deleted to keep the file size under the limit set by BMC bioinformatics). [file 1471-2105-5-147-S2.bz2 › chilibotAdditionalFile2/dip50/204ID8915609E697/html/APP_TTR.html]

 


 **APP** and **TTR** 
  
Found 112 abstracts in PubMed, retrieved 50.  
 

 What does Google say? 
 PDF only 
| .edu only 

---

**Interactive relationship** (e.g. stimulation, inhibition, etc)

**Inhibitory relationship**- The amyloidogenic processing of  **APP**  can be enhanced by apolipoprotein E, reduced by transthyretin  [ **TTR** ] , and modulated by several cytokines.  Ref: 10818483 Ann N Y Acad Sci, 2000
- **APP**  5 UTR conferred translation was selectively down regulated in response to intracellular iron chelation using three separate reporter assays chloramphenicol acetyltransferase  [ **TTR** ] , luciferase, and red fluorescent protein reflecting an inhibition of  **APP**  holoprotein translation in response to iron chelation.  Ref: 12198135 J Biol Chem, 2002
- The amyloid beta  [ **APP** ]  25 35 at a dose of 5 or 15 nmol rat impaired radial arm maze performance, and induced a decrease in choline acetyltransferase  [ **TTR** ]  activity in the medial septum, cortex and hippocampus, but not in the basal forebrain.  Ref: 11166290 Eur J Pharmacol, 2001
- The expression of transthyretin  [ **TTR** ] , a protein shown to sequester Abeta and prevent amyloid fibril formation in vitro, and several genes in the insulin signaling pathway, e.g., insulin like growth factor 2, were increased selectively in the hippocampus of  **APP**  mice.  Ref: 12196559 J Neurosci, 2002
**Neutral relationship**- **TTR**  is an amyloid beta  [ **APP** ]  protein scavenger, so an increase in its expression could prevent amyloid aggregate formation.  Ref: 12566565 Proc Natl Acad Sci U S A, 2003
- Transthyretin  [ **TTR** ]  binds amyloid beta  [ **APP** ]  peptides, Abeta1 42 and Abeta1 40 to form complex in the autopsied human kidney possible role of transthyretin  [ **TTR** ]  for abeta sequestration.  Ref: 10704770 Neurosci Lett, 2000
- Using the coupled assay method for thioltransferase  [ **TTR** ]  activity, GPX had a kcat  **app**  of 186 9 min 1 and specificity constants  **app**  of 1.  Ref: 10198252 Biochem Biophys Res Commun, 1999
- We now demonstrate that the cytoplasmic tail of  **APP**  forms a multimeric complex with the nuclear adaptor protein Fe65 and the histone acetyltransferase  [ **TTR** ]  Tip60.  Ref: 11441186 Science, 2001
- Transthyretin  [ **TTR** ]  plays a role in hormone transport in the brain and possibly a neuroprotective role by amyloid beta  [ **APP** ]  sequestration.  Ref: 11381109 Proc Natl Acad Sci U S A, 2001
- Surprisingly, KAI1 is also directly activated by a ternary complex, dependent on the acetyltransferase  [ **TTR** ]  activity of Tip60, consisting of the presenilin dependent C terminal cleavage product of the amyloid beta precursor protein  [ **APP** ]   **APP** , Fe65, and Tip60, identifying a specific in vivo gene target of an  **APP**  dependent transcription complex in the brain.  Ref: 12150997 Cell, 2002
- The amyloid precursor protein  **APP** , a large glycoprotein highly expressed in neurons, is cleaved in its intramembranous domain by gamma secretase to generate amyloid beta  [ **APP** ]  and a free carboxyl terminal intracellular fragment  **APP**  CT, which has previously been suggested to interact with the adapter protein Fe65 and the histone acetyltransferase  [ **TTR** ]  Tip60.  Ref: 12032152 J Biol Chem, 2002
- Cultured muscle fibers CMF from a patient with inclusion body myositis IBM and cardiac amyloidosis associated with the transthyretin  [ **TTR** ]   **TTR**  Val122Ile mutation contained aspects of the IBM phenotype vacuolation, congophilic inclusions, and clusters of immunocolocalizing amyloid beta  [ **APP** ]  peptide Abeta and  **TTR**  accumulations.  Ref: 12874414 Neurology, 2003
- In addition, the increased expression of transthyretin  [ **TTR** ]  and insulin like growth factor 2 and the increased phosphorylation of Bad in hippocampal neurons were maintained in 12 month old  **APP**  mice when compared with age matched controls.  Ref: 12196559 J Neurosci, 2002

**Non-interactive relationship** (e.g. studied together, co-existance, homology, etc.)

- **APP**  C58 induced apoptosis was markedly enhanced by co transfection with wild type Tip60 and decreased by mutant Tip60 lacking histone acetyltransferase  [ **TTR** ]  activity, suggesting that Tip60 mediates  **APP**  CT induced cell death.  Ref: 12032152 J Biol Chem, 2002
- We found that the transthyretin  [ **TTR** ]   **TTR**  gene, whose product has been shown to bind to amyloid beta  [ **APP** ]  Abeta protein and prevent Abeta aggregation, was more abundantly expressed approximately 1.5 to 2.0 fold in the brainstem and hippocampus areas containing choroid plexus of nicotine SA rats.  Ref: 10662821 J Neurosci, 2000
- Effects of aging and amyloid beta  [ **APP** ]  peptides on choline acetyltransferase  [ **TTR** ]  activity in rat brain.  Ref: 11958528 Neurochem Res, 2002
- Transient transfection of astrocytes with  **APP**  gene promoter chloramphenicol acetyltransferase  [ **TTR** ]  reporter constructs led to increased reporter activity upon TGF beta stimulation.  Ref: 12099697 Biochem Biophys Res Commun, 2002
- Analysis of  **APP**  promoter activity upstream of the chloramphenicol acetyltransferase  [ **TTR** ]  reporter gene in normal human astrocytes, revealed that the  **APP**  promoter binding beta site is responsive to TGF beta.  Ref: 12099698 Biochem Biophys Res Commun, 2002
- A transcriptionally correction of transcriptively active complex of  **APP**  with Fe65 and histone acetyltransferase  [ **TTR** ]  Tip60.  Ref: 11441186 Science, 2001
- Effects of amyloid beta  [ **APP** ]  25 35 on passive avoidance, radial arm maze learning and choline acetyltransferase  [ **TTR** ]  activity in the rat.  Ref: 11166290 Eur J Pharmacol, 2001
- Phosphorylation of 69 kDa choline acetyltransferase  [ **TTR** ]  at threonine 456 in response to amyloid beta  [ **APP** ]  peptide 1 42.  Ref: 12486117 J Biol Chem, 2003
- Several cerebrovascular amyloid proteins amyloid beta  [ **APP** ]  protein Abeta, cystatin C ACys, prion protein AScr, transthyretin  [ **TTR** ]  ATTR  [ **TTR** ] , gelsolin AGel, and ABri or A WD have been identified, leading to the classification of several types of CAA.  Ref: 10935432 Neuropathology, 2000
- MATERIAL AND METHODS  **APP**  prealbumin  [ **TTR** ] , albumin, transferrin, haptoglobin were measured by automatic kinetic analyzer JCS II TM Beckman in 92 patients with acute and 61 patients with chronic viral hepatitis.  Ref: 11270948 Ter Arkh, 2000
- Our data suggest that the choroid plexus cells produce molecules involved in processes such as prevention of fibrillization of amyloid beta  [ **APP** ]  protein transthyretin  [ **TTR** ]  and gelsolin, lipid metabolism phospholipid transfer protein and ABCA8, and detoxification androgen inducible aldehyde reductase.  Ref: 12614681 Neuroscience, 2003
- Hence, we performed multiple labeling immunocytochemistry for choline acetyltransferase  [ **TTR** ]  ChAT, neuron specific nuclear protein NeuN and  **APP**  4, and 48 h after NMDA infusion in the MBN.  Ref: 11997684 Neuroreport, 2002
